# A Safe Protocol for Amalgam Removal

**DOI:** 10.1155/2012/517391

**Published:** 2012-01-18

**Authors:** Dana G. Colson

**Affiliations:** Dr. Dana Coloson & Associates, 1950 Yonge Street, Toronto, ON, Canada M4S 1Z4

## Abstract

Today's environment has different impacts on our body than previous generations. Heavy metals are a growing concern in medicine. Doctors and individuals request the removal of their amalgam (silver mercury) restorations due to the high mercury content. A safe protocol to replace the silver mercury filling will ensure that there is minimal if any absorption of materials while being removed. Strong alternative white composite and lab-processed materials are available today to create a healthy and functioning mouth. Preparation of the patient prior to the procedure and after treatment is vital to establish the excretion of the mercury from the body.

## 1. Introduction

In dentistry, there is a lot of controversy about the topic of silver mercury fillings; are they safe or not safe? There are many articles written on the pros and cons of these types of fillings. It is difficult to quantify and to assess the effects in each individual. It is not easy to identify silver mercury fillings as the cause if illness presents or if the fillings contributed to illness, except in extreme toxicity cases. Refer to the beginning sections of this review paper concerning the science and mechanism of how mercury interconnects with body tissues and functions.

Environmental doctors investigate heavy metal toxicity as part of their overall wellness regiment to help their patients with health concerns. These doctors look at sources of metals when the patient's lab reports/diagnostic tests show high levels of mercury and other metals. They investigate what sources are contributing and how to reduce the burden on the body. The doctor may prescribe the safe removal of silver mercury fillings so as not to create an additional burden on the body and to help their patient heal. Thus, when removing amalgams, additional steps help ensure that the patient is protected.

## 2. Introduction of Amalgam in Dentistry

Dental amalgam restorations, also called silver mercury fillings, were introduced to North America in the 1830s and have been the standard restorative filling for our molars and premolars. At that time there was a lot of controversy about its intraoral use. Silver mercury fillings began to take over the cast gold and gold foil restorations. These were excellent and lasted for years; however they were labour intensive and the cast gold required a lab process that centrifuged gold into a wax pattern to fit the tooth accurately. This was a two-appointment process with added expense. Gold foil restorations were often traumatic to the pulp of the tooth, creating necrosis and requiring root canal. The addition of amalgams as a restorative filling was a welcomed opportunity to offer at a substantial cost reduction as the mercury was triturated with a pellet containing silver, copper, tin, and zinc. This created a substance that could be placed into the cleaned out tooth structure where decay had been present. It was packed, condensed, and allowed to harden within a few minutes and then carved intraoral chairside. Today the extra, unused amalgam is placed in a container for safe disposal. This restoration is easily burnished to tooth structure to recreate the tooth to its original shape and size. The onset of amalgam allowed people to keep their teeth, rather than having them extracted if money did not allow for gold restorations. Keeping teeth enabled people to have better digestion and supported a more balanced quality of life.

Today, with the increase of chemicals such as pesticides, preservatives, processed ingredients in food, and diverse contaminants in our environment; sensitivities, allergies, and other illnesses are increasing rapidly. *The Brain Wash* postulates that the toxins in our society are not additive but synergistic. For example, the average apple contains residue of eleven different neurotoxins and is sprayed with pesticides seventeen times prior to being picked from a tree [1]. Our food intake of many pesticides and additives is most often unknown. The level of materials such as mercury that our bodies could tolerate several decades ago may not be what we can sustain today.

## 3. Amalgam and Composite Fillings

Silver mercury amalgam restorations are comprised of 50% mercury, with the balance being silver, copper, tin, and zinc [2]. Over time the exposed surface changes. The fillings corrode, and surface texture becomes rough. People who chew gum create a smooth, shiny surface on their fillings. Mercury vapor is released by chewing grains, nuts, seeds, and gum, as detected using mercury vapor analyzers [3]. A study in 2010 looked at the wearability of composite (white) restorations compared to amalgams. It showed that over 12 years, the group of patients that were not prone to decay, with resin/composite-filled restorations, were better off than the group of patients with silver amalgam restorations [4]. Today with awareness of diet, home care, and education, the majority of people who seek preventative dental care are less prone to decay. The author has worked with alternative restorations for over 27 years.

The advantage of white composite restorations is that composite binds to composite and the base of the tooth rarely needs to be disturbed once the amalgams are removed. Dental restorative materials have various components, and individual Material Safety Data Sheets (MSDSs) are available from the manufacturer. If an individual has concerns or is sensitive to materials, one can refer to these reference sheets. For example, there are many composites and bonds available today without bisphenol A. Psychological benefits are also a positive factor for patients. People feel that they now have a mouth without the “scars” of the past. They are no longer self-conscious when smiling, laughing, and singing.

With the introduction of composite restorations, many modifications have been made with the materials and applications due to the extensive ongoing technology and research. The concerns with good marginal seals and prevention of recurrent decay have been diminished. Wear and polishability of the composite materials with nanohybrid particulates can withstand stronger chewing forces. Composites are technique sensitive, and various aids can be used to ensure a proper seal of the restorative material to the tooth structure and to create tight contacts to the adjacent tooth to prevent food impaction between teeth. Today we aim for minimally invasive dentistry to maintain integrity of the tooth structure, and white composite materials are ideal for these restorations.

## 4. Considerations prior to Amalgam Removal

When examining a patient for amalgam removal upon request, many factors must be looked at including the rate of wear/attrition on their teeth, pressures exerted, type of diet consumed on a daily basis, their oral hygiene, and other metals in their mouth. Often amalgam restorations exist under crowns and amalgam tattoos (discoloration along the gum) are noted. Amalgams have also been used to seal the apex of root canal treated teeth. If heavy pressures are exerted by an individual or there is evidence of grinding and clenching, then the longevity of a composite restoration may be compromised. The size of the restoration will also influence the choice of materials. Tooth cusps often fracture over time, as well as with excessive pressure, requiring an indirect restoration to be fabricated by a lab. Today the increasing trend is to work with a computer-generated restoration to secure/repair the tooth in the long term. Bite plates to prevent grinding and clenching help preserve these new restorations from excessive wear and pressure.

When the patient is seen for an initial exam, a thorough medical and dental history is taken. Records including radiographs and intraoral pictures are taken, and a comprehensive exam follows. Previous films are requested or brought in by the patient. Lengthy conversations ensue to make sure that the patient is properly prepared and that we are working with their physician, in a timely manner, to complement the detoxification process that their doctor has prescribed and is administering. The physician evaluates the overall health of the body and the ability of the individual to eliminate toxins. For example, if a patient has a leaky gut, physicians restore this prior to removal as it is difficult to flush out toxins [5]. If a woman is pregnant or breast feeding, amalgam removal does not occur until she has completed breast feeding her child [6]. It has been reported that the mercury concentration in the blood of the fetus can be thirty times greater than the mother's blood [7]. Supplements are helpful and are prescribed on an individual basis by the physician. Vitamin C intake is recommended, often with other supplements, prior to and following amalgam removal. Once the amalgam restorations have been removed, the physician continues to work with the patient to help with the detoxification of mercury that is stored in the body.

## 5. Chairside Procedures

The following steps are taken when removing silver mercury fillings, to ensure minimal if any absorption sublingually, or through the mucosal tissues, and to minimize mercury vapor absorption through the blood/brain barrier [8–10].

In office, the patient is prepared as follows, prior to amalgam removal:

- the patient is draped with a plastic apron under the dental bib to cover their clothing;
- a dental dam (“raincoat”) is customized to fit the existing tooth/teeth to prevent particulates from contacting the oral mucosa;
- underneath the dam, activated charcoal or chlorella is placed, along with a cotton roll and gauze. This helps to intercept particles and to chelate dissolved metals that seep under the dam. Often the particles are found on the sublingual tissues and lateral borders of the tongue. This must be prevented as this is the fastest absorption route into the body;
- the patient's face is draped under the dam, with a liner;
- goggles for the eyes and hair cap or bonnet protection are placed;
- oxygen is supplied to the patient with a nasal mask and the mercury vapor ionizer is turned on. The vapor ionizer is a specialized air filtration system that is used to bind mercury vapors that are attached by the negative ion flow and are then carried to a positively charged ionizer plate at the opposite end of the room.

The operators also protect themselves with a filtered mask, eye and hair protection, and face shields.

The removal of amalgam commences as follows:

- a new dental bur is used in the handpiece to ensure easy removal;
- high volume suction and a continual addition of water spray are supplied to the site where the amalgam is being extracted;
- if possible, the amalgam restoration is sectioned and then scooped out to eliminate as much mercury vapor release as possible [11]. The vitality of the tooth is always a concern and the less trauma to the tooth, the healthier the pulp, which supplies blood vessels and nerve supply to the tooth. The deeper the restoration, the greater the chance of pulpal degeneration, causing necrosis and subsequent abscess at the apex of the tooth, as well as bone loss.

Once the amalgam is removed completely,

- the oxygen and protective coverings are taken away;
- an immediate inspection under the dental dam occurs. The gauze, cotton roll and activated charcoal/chlorella are wiped away. Gauze is then used to inspect the floor of the mouth and tongue to make sure no particulates seeped under the dam;
- once all mucosal tissues are fully inspected and cleaned, the mouth is flushed with copious amounts of water, again to ensure no ingestion or absorption of amalgam particulates.

The tooth is then restored to a healthy state of form and function. Materials are taken into consideration as discussed previously on an individual need. Often environmental healthcare providers give direction on the preferred choice of materials to be used through biocompatibility testing. It is the dentist's ultimate responsibility to advise the patient about the strengths and limitations, if they cannot tolerate some materials. It has been the author's experience that once the amalgam materials have been removed and the patient detoxes under the supervision of their physician, the range and variety of materials increase, allowing the dentist to create the best prognosis for the tooth.

Dentists by law in Ontario [12] and elsewhere in Canada must have a certified amalgam separator on the wastewater lines in dental offices in their practices and must use a certified hazardous waste carrier for the recycling and disposing of amalgam waste. 

## 6. After Amalgam Removal

 A 2011 Norwegian study showed a 3-year followup after amalgam removal with precautions in a treatment group compared to a reference group. It showed significant reductions in intraoral and general health complaints [13].

The following is a list of outcomes that I repeatedly hear from my patients over the years. Although I have not scientifically collected them, after amalgam removal and detoxification, they have also been reported in the literature. Comments include that

- patients no longer have a metallic taste in their mouth;
- patients feel as if they have more energy;
- patients are able to concentrate better and make decisions easier (the “brain fog” is gone);
- their body responds better to other treatments, as if a barrier has been lifted.

To achieve effective results one must include an integrative approach with a physician and health care team with attention to detoxification and diet over several months, with laboratory tests to monitor progress.
